# Beliefs that contribute to delays in diagnosis of prostate cancer among Afro‐Caribbean men in Trinidad and Tobago

**DOI:** 10.1002/pon.5085

**Published:** 2019-04-29

**Authors:** Michelle King‐Okoye, Anne Arber, Sara Faithfull

**Affiliations:** ^1^ Faculty of Health and Medical Sciences University of Surrey Guildford UK

**Keywords:** Afro‐Caribbean men, experiences, oncology, prediagnosis, prostate cancer, Trinidad and Tobago

## Abstract

**Objective:**

The aim of this study was to explore Trinidad and Tobago (TT) men's prediagnosis experiences of prostate cancer (PCa). This study is part of a wider project that examined men and their partners' experiences of routes to diagnosis for PCa in TT.

**Methods:**

Men (n = 51) were voluntarily recruited to semi‐structured interviews from four centres. Data were analysed following principles of grounded theory.

**Results:**

Major barriers to medical help seeking were highlighted as lack of knowledge and awareness of the prostate gland and symptoms of PCa, the digital rectal exam (DRE), prostate‐specific antigen (PSA), cultural and religious beliefs, and hegemonic masculinity norms and nonreporting of bodily changes to GPs. Fear of DRE, distrust in providers, and misinterpretation of bodily changes as related to ageing and diabetes mellitus also contributed to delays towards seeking medical help. Men's interactions with pharmacists and traditional healers lengthened the time taken to consult with health care providers for prostate concerns.

**Conclusions:**

TT men's PCa prediagnosis experiences are important to unearth barriers and facilitators to care along routes to diagnosis for this disease. This can help target specific health promotion strategies to motivate men to seek medical care for symptoms in a timely manner.

## 1 BACKGROUND

The Republic of Trinidad and Tobago (TT) has close proximity to South America. It is an ethnically diverse nation including African settlers who originate from slaves during the precolonial era, indentured labourers from East India, British and French settlers, and immigrants from neighbouring countries.^1^, ^2^ Prostate cancer (PCa) is the most common cancer and leading cause of death among Afro‐Caribbean men in TT.^3^ Reports from the National Cancer Registry have shown that men present late with symptoms and are frequently diagnosed with advanced PCa.^4^, ^5^ Some reasons for being diagnosed with advanced PCa were lack of PCa screening programmes, low screening uptake, and inadequate cancer care services.^4^, ^5^ There is a dearth of explorative research surrounding TT men's PCa prediagnosis experiences. A recent systematic review highlighted how men's beliefs and perceptions of prostate symptoms were linked to their interpretation of symptoms and the meanings attached to these and associated help‐seeking actions.^6^

### 1.1 Aim, objective, and research questions

The aim of this article is to report on TT men's prediagnosis experiences of bodily changes and symptoms to unearth their beliefs and perceptions about their illness along routes to diagnosis of PCa. The research questions were as follows: “What are TT men's prediagnosis experiences of PCa?” “What are men's beliefs and meanings about PCa?” “What beliefs and interpretations guided men's help‐seeking for prostate symptoms?”

### 1.2 Methodology

Grounded theory (GT) was deemed appropriate for this study as very little was known about why TT men present with symptoms of advanced PCa and meanings they associate with their bodily changes. GT enables the researcher to capture psychosocial thoughts and processes of behaviour to explain “how” and “why” men behave in a particular way regarding their experiences of symptoms leading up to diagnosis.^7^ This study utilized a Straussian GT approach, which employs symbolic interactionism (SI) and considers the micro and macro as well as broader contextual factors (socio‐economic, political, cultural, and religious) that may influence the phenomena under examination.^7^ This enabled the research to include multiple perspectives as well as the social processes governing behaviour to be unearthed. GT is an inductive process that facilitates an unravelling of participants' experiences and enables theory development that is representative of participant's experiences.^7^ Reflexivity is embedded in SI, which enables the researcher also to reflect on her assumptions and personal biases and transparency of research procedures throughout the trajectory of the study.^8^

### 1.3 Ethical approval and considerations

Ethical approval was obtained from University Ethics Committee, University of Surrey (EC/2014/91/FHMS) and by each participating regional health authority (RHA) in TT (North West, North Central, and Division of Health and Social Services). Participants' identities were protected by use of pseudonyms. Institutions were also anonymised to maintain confidentiality.

### 1.4 Recruitment and data collection

As the study purpose was to explore men's experiences leading up to diagnosis with PCa, it was critical to target those diagnosed with this disease. Therefore, urology and oncology centres in Trinidad (n = 2) and Tobago (n = 2) were targeted to recruit men recently diagnosed with PCa.

A research flier was utilized at centres to recruit potential participants to the study. Men identified with PCa were approached with the help of oncology staff using the participant information sheet. Once a man was identified as interested in the study, he was approached by M.O. and invited to participate and any questions answered by the researcher. Written informed consent was obtained from all participants. Interviews were conducted at the hospital centre based on patient preferences.

M.O., the primary researcher, an Afro‐Caribbean researcher in her thirties, has a background in cancer care and experience in conducting qualitative research. M.O. conducted and transcribed all interviews at the four centres within both islands of TT in 2015 and 2016. M.O. was also culturally competent towards understanding this population having previously lived and worked in TT. Table S1 shows the eligibility criteria. It was thought important to capture all representations of ethnicities from Afro‐Caribbean men in TT as outlined in Table S1.^2^ Men from other ethnic groups, Indo‐Caribbean (East Indian ancestry) and Chinese, were also included in the study. This achieved a diverse and heterogeneous sample as shown in Table S2. Demographics details were obtained prior to the conduct of interviews. All interviews were audio‐recorded and transcribed by M.O.

## 2 METHODS

Face‐to‐face semi‐structured interviews guided by a topic guide were used to explore men's beliefs and meanings of their journey to diagnosis enabling flexibility towards their experiences through narratives and in‐depth accounts of their experiences.^9^ These enabled the researcher to engage with participants and build rapport while observing facial expressions, tone of voice, and body language. The interview commenced by asking an open question to build rapport, “Can you tell me a little of yourself?” This enabled participants to be comfortable before getting into the body of the interview. The question “Can you share with me your story as to how you came to where you are now?” facilitated men's report of their illness journey as they experienced it. Using theoretical sampling, initially three interviews were conducted with men and transcribed. Concepts derived from these interviews guided further data collection, which was continued throughout the study. Apart from ethnicity, other variables considered during theoretical sampling are also outlined in Table S1. Theoretical saturation was achieved with 25 men in the first island of Tobago. Thereafter, in order to achieve a comparative sample in Trinidad, similar numbers and characteristics of men were interviewed in this island.

## 3 DATA ANALYSIS

Interviews were transcribed verbatim. Data analyses and further data collection were guided utilizing the principles of theoretical sampling and constant comparison. M.O. and A.A. checked transcriptions. Data was verified, analysed, and interpreted independently and collaboratively by all researchers throughout the research trajectory. Memo writing and the use of a reflexive journal were employed throughout the study. Nvivo 10 coding software facilitated coding and retrieving. Following analytic processes such as open coding, axial coding, and selective coding procedures,^7^ it emerged that the men described a process of normalisation of their symptoms and actions to self‐manage and hide their bodily changes. Current and relevant literature was employed simultaneously with data analysis, which facilitated theoretical sensitivity. Theoretical saturation was achieved as no new data were emerging.^7^

## 4 RESULTS

Participants were diagnosed with PCa, aged between 42 and 90 years, and from varied ethnicities, religious, and socio‐economic backgrounds (see Table S2). Themes generated from the data are discussed as (a) beliefs and meanings about PCa and normalisation of symptoms, (b) taboos and sensitivity in the experience of bodily changes and body invasion, and (c) use of herbs: “I managed it myself.” Themes represent trends and patterns across the sample. Table S4 shows help‐seeking delays with sample excerpts and number of men with these experiences.

### 4.1 Beliefs and meanings about PCa and normalisation of symptoms

Men reported varied beliefs about the normality of the symptoms they experienced. Poor urine flow and poor stream were associated with older age. Paul reports historically that problems with urination were referred to as “stoppage of water” for those living in the country. Ron felt his urinary dribbling was related to his drinking of tap water. These beliefs were reported to be due to lack of knowledge of the prostate gland:

Long ago they used to call it stoppage of water. Now I know that it's prostate cancer. Like, you can't pass water … when it slowing down. The people call it that … especially those that live in the countryside. People believe that stoppage of water is normal. That it comes as the body ages. If they know that it is cancer they will realize how serious it is and do something about it. (Paul, 72, African settler, farmer, Tobago)

I noticed my urine was slowing down and it started dripping. I felt it had to do with how I was drinking tap water. I didn't know about the prostate gland at that time. (Ron, 65, East Indian, labourer, Trinidad)

Paul identifies that older people normalised urine problems, a problem associated by Paul with getting older, and offers his justification for why those living in rural settings do not seek help with urinary problems. As men age, they are known to suffer benign prostatic hypertrophy leading to poor urinary flow and urinary retention and not necessarily through being a symptom of PCa, and as identified by Paul, if they knew of this link between “stoppage of water” and its seriousness as suggestive of cancer, they would certainly do something about it.^10^ Similarly, Ron normalized his urinary dribbling to his tap water consumption as he lacked knowledge of its association with the prostate gland. This contributed to help‐seeking delays.

Some of the men (n = 32) in the study who suffered from comorbidities, such as diabetes and other past illnesses/injuries, normalised their urinary changes and back pains as related to these rather than the prostate gland.

I had slipped and fall sometime ago so when my back started paining me I felt it had something to do with how I fall down. So I didn't worry about the back pains at that time (Colin, 47, East Indian, carpenter, Trinidad)

I'm diabetic and the first thing I noticed is that you can't hold your pee or anything like that. Also, for some months now I have erectile problems. I felt these were connected somehow with my sugar … but it ain't really bothering me to say I passing blood or peeing blood or anything like that you know … no pain. (Matt, 55, African settler, driver, Tobago)

Colin felt that his back pains were as a result of a previous fall/injury. Likewise, Matt connected his symptom experiences with normal pathology related to diabetes mellitus (DM), where there are also urinary changes and problems holding one's pee. He described how he did not pass blood in his urine nor experienced pain, which are considered alarm symptoms requiring urgent medical care and would legitimate a need to see a health care practitioner (HCP).^11^

Glen and Alex made connections with their cancer as God's doing.

I am a firm believer in God. He allows cancer because there is nothing that God is unaware of. I believe he allows people to get it so they can experience his healing power. For others it might be for spiritual growth. I have witnessed healing in my life on many occasions. (Glen, 72, African settler, engineer, Trinidad)

I believed that this sickness was a sign for me to get right with God. I put off my baptism for a long time now and I realized that was what I needed to do. So I went and get baptized. (Alex, 71, African settler, labourer, Tobago)

Both Glen and Alex felt that their diagnosis of cancer was for the purpose of spiritual growth. Glen perceived this as a test from God and spoke of his prior experiences of divine healing. Alex sensed that he needed to get baptized and pursued it. As such both men were not overly concerned in consulting a doctor as they indicated that their illness was part of God's plan, which indicates an extrinsic locus of control.^12^

### 4.2 Taboo and sensitivity in the experience of bodily changes and body invasion

Kevin and Rick delayed help seeking because of the sensitivity surrounding their bodily changes.

Not really, my erectile problem doesn't bother me you know. I've had it for years and I just kept it to myself. Eventually my wife found out but I never said anything to my doctor. You see men wouldn't want to talk about these things just like that. They would prefer to battle it on their own. It could be shame but I'm not ashamed anymore. I deal with it. This is the first time I'm talking about this actually. (Kevin, 46, African settler, labourer, Tobago)

I had a burning in the penis when I went to pass water. I'm a private person. I don't like stripping down in front of anybody. That sort of kept me from telling the doctor about my problem in the first place. (Rick, 50, African settler, carpenter, Trinidad)

This was the first time (in the interview) Kevin spoke about his erectile problems. Prior to this, Kevin kept this secret because of feelings of shame. He stated that men “battle” with bodily changes on their own because they regarded these matters as sensitive. Similarly, Rick did not tell his doctor about his urinary changes because of his private nature. The sensitive nature of their symptoms contributed to help seeking delays. Bury^13^ highlights how hiding symptoms from others is one main feature that occurs at the initial stage of the symptom experience, which can be effective in hiding illness but becomes limited when the problem is eventually inevitable through being recognized by others. Keeping symptoms secret enables individuals to make sense of their bodily changes and to try strategies to adapt to the situation. Kevin now feels able to talk about his impotence openly and without feeling shame. Kevin stated that he did not discuss his erectile changes with his wife at the initial stages. However, he reported that she eventually found out as time progressed and he could no longer hide his problem.

Leo and Adam describe their stoicism in managing pain for years before being diagnosed with advanced PCa:

I had pain in all my joints from the waist go down … for about two years now before I went to see the doctor. As a man you know I could bear pain. I used to ask the pharmacist off and on for any tablet that could help me with the pain … because at times I couldn't even go to work. Sometimes the stronger tablets would keep the pain down for a short while but it didn't last for long so I would just pop in to the pharmacy and get something to keep me up. (Leo, 61, Mixed, business owner, Trinidad)

This back pain was terrible. I took painkillers for years so I could work and support my family … but it didn't work. I had to see a doctor when I couldn't bear it no more. (Adam, African settler, 53, labourer, Tobago)

Both men bore their pains in order to work and support their families. Their self‐resilience and stoicism are connected to hegemonic masculinity. These men reported taking over‐the‐counter (OTC) analgesia to manage their symptoms. Leo describes how he is managing his pains “as a man,” in which men perceive that they should be macho, strong, and self‐resilient. Leo's pharmacist sanctioned his continued use of OTC analgesia, which hindered his medical help–seeking actions. This finding was similar to a study that identified a culture of masculinity existed among men in which they were expected to conform to societal norms.^14^

Bearing pains due to stoicism and perceptions that Black men are stronger compared with other ethnicities were reported. This was reflected in Alan's and Tim's experiences.

At first I couldn't understand how this could happen to me, a strong Black man. That's how we African men see ourselves. We tell ourselves we are strong and could never get sick until something happen. I never went to any doctor for my whole life until this. (Tim, 64, African settler, Retired police officer, Tobago)

I think we sometimes feel that we Black men are strong. At least that's what slavery taught us. So society expects us to bear our pains and our health problems. Maybe, that's why we don't share how we feel even when we are really ill. What would people think? Nobody bothers about the Indians, the Chinese, and the Spanish men. Those men would bawl and carry on and nobody thinks anything of it. But when we African men go to the hospital for a problem we get everybody watching us … doctors and nurses too. They're watching us in a funny way. (Alan, 66, African settler, retired teacher, Trinidad)

Tim demonstrated perceptions that African men are strong and not susceptible to illness. Alan's perceptions were similar. However, he also highlighted differences in cultural beliefs and why TT men are unable to share their feelings. Alan believed that African men were deterred from showing weakness and sharing feelings about ill health because of societal norms originating from slavery, which deemed them stronger than other ethnic groups in TT. He stated that while men from other ethnic groups were free to express their vulnerability, men of African descent felt hindered from doing so because of them being watched and observed by doctors and nurses. This aspect of surveillance was introduced by Ragsdale as a stereotypical threat that can deter nondominant groups' behaviours.^15^ Alan's experiences are also supported by a study that found racial bias among laypeople and HCPs' beliefs towards black men's higher ability to bear pain. Some were based on perceptions of the black men's skin being thicker than other ethnic groups.^16^ These are evident in Alan's narrative about the Spanish, Chinese, and Indians expressing distress, which would not be relevant to a man of African heritage as stoicism was the way of coping with distress.

Some men in the study like Dereck held the belief that doing “the finger test” (digital rectal examination [DRE]) was equal to being homosexual. This belief promoted fear in engaging in early PCa screening and contributed to delays. Other men like Trevor were unaware that tests were available for PCa, including the health services that offered these.

The finger test is a no no for me. I don't believe that men should go there. I feel that if I do that test people will think I am a homosexual. That makes me feel uncomfortable. (Dereck, 51, Health officer, Dougla, Trinidad)

I never heard of any test for prostate cancer. Do they do that in Trinidad? I have no idea about any test here. Never heard of any. (Trevor, 67, Mixed, Pensioner, Trinidad)

Dereck voiced feeling uncomfortable about the nature of the DRE test. His belief was influenced by societal norms, as he was concerned about what people might say about him. Trevor, on the other hand, was not aware of tests being offered by health services for detection of PCa. Although men from higher socio‐economic backgrounds expressed these views, they still participated in screening because they understood the importance of this.

### 4.3 Use of herbs: “I managed it myself”

Most men were very keen to manage their bodily changes themselves and did not easily identify problems that needed immediate medical care. To a large extent, they avoided contact with health services. Men felt that using herbs for health problems was a more natural and safer approach than conventional medicine.

I've been sick before and I managed it for myself and it worked. Herbs are safer and more natural. I've used herbs that I boiled and drink for headaches and the cold. Medications have too much side effects. Some people doctors diagnose, and doctors are not perfect and they may diagnose cases and might not diagnose correctly and patients pass away. I know people personally who died at the hands of doctors. (Baxter, 70, African settler, labourer, Tobago)

I believe God put things in motion. Natural food is better, like vegetables, herbs and that is how the world going back to what people used in the past. Like there are natural herbs that could cure prostate cancer‐like fitweed and ciprium. I used these herbs when I started having problems with my urination. I still use it. I heard some men got healed from some of these herbs. (Tony, 45, Mixed, carpenter, Trinidad)

Baxter's belief was based on accounts of others that died at the hands of doctors. He felt that the side effects of medications posed a threat to the body. He believed in herbal use for health concerns. He also demonstrated a lack of trust in doctors' ability to diagnose accurately by giving accounts of persons he knew that died as a result of medical negligence. Tony identified herbs that other men used that were effective towards treating PCa, which influenced him to use it for his symptoms. For some men, their usage of herbs lengthened the time taken to see a doctor.

Some men reported visiting the traditional healer (TH) when their symptoms of urinary burning and dribbling and back pains continued.

That happened a long time ago when I first had the problem with peeing. I didn't know what was causing it so I went for jharay. I tried that first, but the problem continued so I had to come here for the doctor to see what happening with me. I couldn't pee. (Seth 62, East Indian, labourer, Trinidad)

I went and he rub me down, he did his thing. It worked for a while. My back pain had gone for months but then it came back. It is a very nasty disease. (Alex, 71, African settler, labourer, Tobago)

Seth's and Alex's visit to the TH was related to their cultural and religious practices. They turned to religious leaders in the midst of uncertainty about bodily changes. Most men in the study reported frequent church attendance, prayer requests, and Bible study engagement for healing of their symptoms. The role of religion during symptom experiences was instrumental towards physical and emotional healing, a coping mechanism and provision of hope for patients. However, for some men, this lengthened the time taken to see a doctor for prostate symptoms.

## 5 DISCUSSIONS

Men experienced many bodily changes (Table S3). Red flag symptoms such as blood in urine and urinary retention facilitated help seeking within 1 to 7 days. Men that experienced other bodily changes such as joint pains, numbness and pins/needles in extremities, dribbling, straining, and urgency to urinate took an average of 3 to 6 months to seek medical help. Some men took up to 2 years to seek help for back and groin pain, fatigue and weight loss, and nocturia, and some never reported symptoms of erectile dysfunction to their GPs. Barriers to medical help seeking are outlined in Table S4.

TT men shared how their beliefs and meanings about bodily changes were complex, as well as their reluctance to identify illness and how they self‐managed their bodily symptoms, which were often severe and considered alarm symptoms.^17^ They made links between urinary problems and ageing and in so doing normalised their urinary problems as something to be expected as you age. They also made connections with other illness they had such as DM and made their own links between urinary changes and DM rather than prostate‐related symptoms. These findings resonate in other studies that highlight legitimization of prostate bodily changes as major barriers to medical help seeking.^18^, ^19^, ^20^

The findings also demonstrate the taboo nature of symptoms that would reveal men's vulnerability and threaten their manhood, hence their approach to both conceal and self‐manage symptoms as much as possible. This highlights the role of masculinity and stoicism among Afro‐Caribbean men, which aligns with the cultural context of TT. These findings mirror another study, which examined men's first year following diagnosis of localized PCa, where men were found to hide their emotional distress by not talking about their feelings and avoiding conversations that may lead to these.^21^ However, the current study differs in that most men interviewed were diagnosed with advanced and late‐stage PCa. It was also found there was a taboo related to the DRE due to its connotations with homosexuality, which was also found in Ocho and Green's study among men from the general population in TT.^22^

The use of OTC drugs to manage prostate symptoms among men already diagnosed with PCa were found in medical literature, which supports the current study.^23^, ^24^ Men's interactions with pharmacy staff for OTC medications during the prediagnosis phase was highlighted in this study. Use of pharmacy services for self‐management of symptoms has been recorded among the general public.^25^ Since pharmacy staff and pharmacists were stated as first port of call for managing bodily changes in the current study, they can be targeted as a nonthreatening way for promoting awareness of symptoms connected to prostate disease in a timelier way as the study identified that the average time that it took men to reach a doctor with symptoms of PCa was 6 months.

Men's use of THs were reported in this study as delaying medical help seeking for prostate symptoms and are rooted within the cultural beliefs of TT. This was echoed in O'Brien's study in which men with symptomatic presentation of PCa consulted with THs for healing, which delayed medical help seeking.^26^ In the current study, religion served a pivotal role towards men's PCa beliefs. Men either delayed help‐seeking because of their belief that God will heal them or attended health services because of their belief that God can heal them through doctors' skills and expertise. Similar reports of these are found in Black and Minority Ethnicity (BME) literature.^18^, ^19^ Cultural and religious leaders including THs and churches can be targeted to discuss workshops geared at increasing awareness of the prostate gland and the signs associated with PCa.

## 6 CONCLUSIONS

### 6.1 Study strengths

This study, being the first one to be conducted in TT, can serve as baseline for PCa studies for this nation, within the Caribbean, and among similar ethnic groups of men. The study was conducted among men aged between 40 and 90 years old recently diagnosed with PCa in both islands of TT, which offered a comprehensive understanding of men's experiences.

### 6.2 Study limitations

It was intended to recruit men from public and private health systems in order to obtain a range of experiences. A greater representation of men's prediagnosis experiences arising mostly from public health systems was noted. Capturing more men attending private health systems would have gained a better portrayal of men's experiences from both health systems.

### 6.3 Clinical implications

This study fills the knowledge gap through highlighting the significance of Afro‐Caribbean men's beliefs and experiences towards appraisal of symptoms and different approaches to help‐seeking actions along pathways to diagnosis for PCa in TT. These findings can be transferable to similar populations as they offer unique insight into Afro‐Caribbean men's prediagnosis experiences highlighting facilitators and barriers to PCa care and time taken to seek medical help for serious bodily changes. Lack of knowledge and awareness of PCa, self‐care practices such as OTC medications, use of TH and normalization of symptoms inclusive of the role of culture and religion, and hegemonic masculinity and stoicism were identified as major barriers to medical help seeking. These beliefs and behaviours extended the time to diagnosis and treatment for PCa. The findings of the current study also contribute to public health policy and health promotion. It elucidates the need for mass media awareness raising and health promotion strategies to increase knowledge of PCa among men in TT. These findings can inform stakeholders and HCPs towards decreasing PCa mortality rates and improving the quality of life and survival for men in TT with PCa.

The role of community outreach programmes with emphasis on education has shown significant progress in increasing knowledge and awareness of the functions of the prostate gland and its connection with PCa, symptoms of the disease, and benefits versus harms of screening.^27^, ^28^ This study also underscores the importance of culturally relevant health information to promote awareness among men in TT. For example, societal norms that links with slavery and perceptions that Black men should be stronger caused help‐seeking delays in the current study. Media programmes that contribute to awareness raising are pivotal to help men avoid being diagnosed with late‐stage PCa and to increase survival through timely diagnosis and treatment.

## CONFLICT OF INTEREST STATEMENT

The authors have declared no conflicts of interest.

## Supporting information

Table S1
shows the eligibility criteria for the study and variables considered during theoretical sampling

Table S2
shows characteristics of TT research participants (M = Married, S=Single, D = Divorced, W=Widower/CL = Common Law, SDA = Seventh Day Adventist)

Table S3:
Bodily changes experienced by TT men with sample quotes and time taken to seek medical help.

Table S4:
Barriers to medical help‐seeking for TT participants and number of men that experienced these barriers
