# Supplementary material for: Beliefs that contribute to delays in diagnosis of prostate cancer among Afro‐Caribbean men in Trinidad and Tobago
Source: Psychooncology. 2019 Apr 29;28(6):1321–7. doi: 10.1002/pon.5085 (PMC6617795; doi:10.1002/pon.5085)
Supplement: Supplementary file 1 — Table S1 shows the eligibility criteria for the study and variables considered during theoretical sampling [file PON-28-1321-s001.docx]

| **Inclusion criteria** | **Exclusion criteria** | **Variables considered for theoretical sampling** |
| --- | --- | --- |
| English Language literature | Non-English Language literature | Differing socioeconomic and educational backgrounds and social class |
| Abstract available | No abstract available | Varied geographical locations: rural and urban settings |
| Men diagnosed with PCa | Studies focused on men’s health issues other than for prostate problems | Differing marital status |
| Female partners of men diagnosed with PCa | Male partners, PCa survivors | Varied religious affiliations |
| Men’s experiences of appraisal, help-seeking, diagnosis, and treatment for PCa | Quality of life, intervention studies, end-of-life and palliative care for PCa; developing tools or assessment of tools | Men that participated in screening programmes and those that accessed health services early |
| Men and partners’ accounts | Accounts of health care providers | Varied ethnicities: Afro-Caribbean, Dougla (union of Afro-Caribbean and Indo-Caribbean), Mixed (Union of African and any of these ethnic groups: Chinese, Amerindians, White, Lebanese/Syrian or Latinos) and Amerindian. |
| Studies conducted from 2000-2017 | Studies conducted prior to 2000 | Representations of experiences of varied health systems: public and private |

Supplementary Table 1 shows the eligibility criteria for the study and variables considered during theoretical sampling
