# Supplementary material for: Beliefs that contribute to delays in diagnosis of prostate cancer among Afro‐Caribbean men in Trinidad and Tobago
Source: Psychooncology. 2019 Apr 29;28(6):1321–7. doi: 10.1002/pon.5085 (PMC6617795; doi:10.1002/pon.5085)
Supplement: Supplementary file 2 — Table S2 shows characteristics of TT research participants (M = Married, S=Single, D = Divorced, W=Widower/CL = Common Law, SDA = Seventh Day Adventist) [file PON-28-1321-s002.docx]

| **#** | **Name/Age** | **Marital Status** | **Occupation/Level of education** | **Religion/Ethnicity** |
| --- | --- | --- | --- | --- |
| **Tobago participants** | | | | |
| 1 | Adam, 53 | M | Labourer/No education | Pentecostal/African |
| 2 | Alex, 71 | CL | Gardener/Primary education | Baptist/African |
| 3 | Stan, 68 | S | Labourer/Secondary education | Pentecostal/African |
| 4 | Pete, 77 | W | Retired labourer/Secondary education | Baptist/African |
| 5 | Tim, 64 | M | Retired Police officer/Tertiary education | Catholic/African |
| 6 | Chris, 66 | M | Retired fire office/Secondary education | Pentecostal/African |
| 7 | Luke, 58 | M | Retired police officer/Secondary education | Pentecostal/African |
| 8 | Mark, 68 | CL | Business owner/Secondary education | Catholic/African |
| 9 | Clement, 54 | M | Farmer/Labourer/Primary education | SDA/African |
| 10 | Floyd, 77 | W | Farmer/Primary education | SDA/African |
| 11 | Baxter, 70 | M | Farmer/Primary education | SDA/African |
| 12 | Kinsley, 63 | M | Driver/Secondary education | SDA/African |
| 13 | Dan, 57 | M | Watchman/No education | Catholic/African |
| 14 | Cameroon, 72 | M | Retired Principal/Tertiary education | Catholic/African |
| 15 | Kevin, 46 | M | Labourer/Secondary education | SDA/African |
| 16 | Jason, 60 | D | Labourer/Primary education | SDA/African |
| 17 | Cain, 71 | M | Gardener/Primary education | Christian/African |
| 18 | Pat, 70 | M | Farmer/Primary education | Christian/African |
| 19 | Harry, 87 | M | Labourer (retired), No education | SDA/African |
| 20 | Glen, 72 | M | Separated/Retired engineer | Pentecostal/African |
| 21 | Wes, 77 | M | Farmer/ No education | SDA/African |
| 22 | Paul, 72 | M | Farmer/ Primary education | SDA/African |
| 23 | Greg, 72 | M | Retired health officer/Secondary education | SDA/African |
| 24 | Damien, 58 | M | Labourer/Not stated | SDA/African |
| 25 | Matt, 55 | M | Driver/No education | Christian/African |
| **Trinidad participants** | | | | |
| 26 | Alan, 66 | M | Retired teacher/Secondary education | Pentecostal/African |
| 27 | Dereck, 51 | M | Health officer/Secondary education | Christian/Dougla |
| 28 | Justin, 54 | M | Labourer/Primary education | Catholic/Mixed |
| 29 | Terrence, 77 | M | Retired office worker/Secondary education | Baptist/African |
| 30 | Frank, 64 | M | Retired salesman/Secondary education | Catholic/African |
| 31 | Kane, 65 | M | Retired accountant/Tertiary education | Muslim/Mixed |
| 32 | Nat, 68 | M | Labourer/No education | Pentecostal/African |
| 33 | Adrian, 58 | M | Business owner/Secondary education | Catholic/Dougla |
| 34 | Eric, 80 | M | Retired teacher/Secondary education | SDA/Mixed |
| 35 | Tom, 50 | CL | Labourer/No education | Christian/African |
| 36 | Tyrell, 54 | M | Unemployed/Retired taxi driver, Secondary education | Muslim/African |
| 37 | Tony, 45 | M | Carpenter/Secondary education | Pentecostal/Mixed |
| 38 | Bas, 62 | CL | Retired labourer/Primary education | Pentecostal/Mixed |
| 39 | Trevor, 67 | M | Pensioner/No education | Catholic/Mixed |
| 40 | Chase, 70 | CL | Pensioner, no education | Pentecostal/African |
| 41 | Lee-Ping, 60 | M | Community Activist, Business owner, secondary education | SDA/ Chinese-Mixed |
| 42 | Leo, 60 | M | Business owner, tertiary education | Pentecostal/Mixed |
| 43 | Willo, 70 | M | Pensioner, no education | Christian/African |
| 44 | Randy, 67 | W | Carpenter, private business owner, tertiary education | SDA/African |
| 45 | Clarence, 70 | M | Engineer, private business owner, tertiary education | Pentecostal/Dougla |
| 46 | Colin, 47 | M | Carpenter, secondary education | Hindu/East Indian |
| 47 | Xavier, 59 | M | Private business owner, secondary education | Pentecostal/Mixed |
| 48 | Ronny, 65 | M | Joiner, primary education | SDA/African |
| 49 | Rick, 50 | M | Carpenter, primary education | SDA/ African |
| 50 | Roland, 55 | M | Carpenter, primary education | SDA/East Indian |
| 51 | Seth, 62 | CL | Labourer, primary education | Hindu/East Indian |

Supplementary Table 2 shows characteristics of TT research participants (M=Married, S=Single, D=Divorced, W=Widower/CL=Common Law, SDA=Seventh Day Adventist)
