# Supplementary material for: Beliefs that contribute to delays in diagnosis of prostate cancer among Afro‐Caribbean men in Trinidad and Tobago
Source: Psychooncology. 2019 Apr 29;28(6):1321–7. doi: 10.1002/pon.5085 (PMC6617795; doi:10.1002/pon.5085)
Supplement: Supplementary file 3 — Table S3: Bodily changes experienced by TT men with sample quotes and time taken to seek medical help. [file PON-28-1321-s003.docx]

| **Bodily changes experienced** | | **Participants descriptions** | **Interpretation (s)** | **Average time taken to seek medical help** |
| --- | --- | --- | --- | --- |
| Urinary changes | Blood in urine | I was peeing blood (Frank) | Danger, serious illness | 1-7 days |
|  | Urinary retention | I couldn’t pee and it was painful (Bas) | Physical stress, tap water | 1 – 3 days |
|  | Dribbling | My urine was coming drip by drip (Alex) | Insufficient water intake, stress | 3 months – 6 months |
|  | Nocturia | I was peeing a lot in the night (Mark) | Too much water intake at night, prostate | 6 months – 2 years |
|  | Burning while urinating | I had a burning sensation coming from my penis when I passed urine (Jason) | Infection, insufficient water intake, build-up of acid in the body | 3 months to 1 year |
|  | Straining to urinate | I had to force to pee (Chris) | Insufficient water intake, physical stress | 3 – 6 months |
|  | Urgency in urination | I couldn’t hold my pee (Clarence) | Physical stress, prostate | 3 months – 1 year |
| Pain | Back pain | My back pain was really bad (Randy) | Physical stress, feeling overworked, fall/muscular injury | Up to 2 years |
|  | Bone pains | All my joints were hurting (Matt) | Physical stress, feeling overworked, insufficient vitamin intake | 3 months to 1 year |
|  | Leg pain | I had pain in my legs (Tyrell) | Ageing, physical stress | 3 months to 1 year |
|  | Groin/penile pain | My private part used to hurt real bad (Dan) | Ageing, physical stress | Up to 6 months |
| Sexual changes | Erectile problems | I couldn’t get an erection (Matt) | Diabetes mellitus, physical stress, feeling overworked | 6 months to never reported to doctor |
| Generalized bodily changes | Weakness | My legs were weak (Damien) | Ageing, feeling overworked | Up to 1 year |
|  | Numbness in extremities | I couldn’t feel my legs at some point (Greg) | Ageing, problems with circulation | 3 – 6 months |
|  | Pins and needles in extremities | It felt like some pins sticking me, ‘ginginee’ (Wes) | Problems with circulation, diabetes mellitus | 1 – 6 months |
|  | Unsteady gait/difficulty maintaining balance whilst walking/standing | I had problems balancing while standing and walking (Adrian) | Ageing, feeling overworked, high blood pressure | 3 months – 1 year |
|  | Loss of control of bowel movement (prolonged constipation) | I felt like something was blocking my passageway. I couldn’t pass stool (Tony) | Insufficient water intake | 1 - 2 years |
|  | Weight loss | I lost a lot of weight (Nat) | Lifestyle changes | Up to 2 years |
|  | Fatigue | I was feeling so tired all the time (Chris) | Feeling overworked | Up to 2 years |

Supplementary Table 3: Bodily changes experienced by TT men with sample quotes and time taken to seek medical help.
