# Supplementary material for: Beliefs that contribute to delays in diagnosis of prostate cancer among Afro‐Caribbean men in Trinidad and Tobago
Source: Psychooncology. 2019 Apr 29;28(6):1321–7. doi: 10.1002/pon.5085 (PMC6617795; doi:10.1002/pon.5085)
Supplement: Supplementary file 4 — Table S4: Barriers to medical help‐seeking for TT participants and number of men that experienced these barriers [file PON-28-1321-s004.docx]

| **Barriers to medical help-seeking** | **Example: Quote from participant** | **Number of participants that experienced these barriers from sample** |
| --- | --- | --- |
| Lack of knowledge | *I felt it had to do with how I was drinking tap water. I didn’t know that it had something to do with the prostate gland at that time (Bas)* | n=40 |
| Hegemonic masculinity norms | *As a man you know I could bear pain (Leo)* | n=45 |
| Self-care | *This back pain was terrible. I took pain killers for years so I could work and support my family (Adam)* | n=47 |
| Herbal usage | *Natural food is better, like vegetables, herbs and that is how the world going back to what people used in the past. Like there are natural herbs that could cure prostate cancer- like fit weed and ciprium. I use these herbs (Stan)* | n=35 |
| Lack of trust in HCPs | *…Doctors are not perfect and they may diagnose cases and might not diagnose correctly and patients pass away (Baxter)* | n=30 |
| Lack of awareness of health-services | *‘I never heard of any test for prostate cancer. Do they do that in Trinidad? (Adrian)* | n=40 |
| Non-reporting of bodily changes to GP | *...My erectile problem doesn’t bother me you know. I’ve had it for years and I just kept it to myself… I never said anything to my doctor (Rick)* | n=30 |
| Co-morbidities/previous health problems | *...That you can’t hold your pee or anything like that. Also, for some months now I have erectile problems. I felt these were connected somehow with my sugar (Matt)* | n=32 |
| Misinterpretation of bodily changes | *My back started paining me I felt it had something to do with how I fall down (Alex)* | n=35 |

Supplementary Table 4: Barriers to medical help-seeking for TT participants and number of men that experienced these barriers
